# A Patient with Eight Intracranial Aneurysms: Endovascular Treatment in Two Sessions

**DOI:** 10.1155/2016/9637905

**Published:** 2016-09-07

**Authors:** Erol Akgul, Hasan Bilen Onan, Huseyin Tugsan Balli, Nuri Eralp Cetinalp

**Affiliations:** ^1^Radiology Department of Medical Faculty, Cukurova University, Adana, Turkey; ^2^Neurosurgery Department of Medical Faculty, Cukurova University, Adana, Turkey

## Abstract

The frequency of multiple intracranial aneurysms seen in patients with or without subarachnoid hemorrhage is high. The advancement of the endovascular technique and devices has ensured that endovascular treatment of intracranial aneurysms is the first choice in most cases, especially in unruptured ones. Different combinations of treatment modalities and techniques can be used in the management of multiple aneurysms. But in selected patients without subarachnoid hemorrhage, treatment of all aneurysms in one or more sessions with endovascular techniques is less traumatic than that with surgery. In the literature, the maximum number of aneurysms in one patient treated endovascularly and/or surgically is seven. In this case report, we present, with a review of the literature, a patient with eight intracranial aneurysms, all of which were treated in two sessions with various endovascular techniques. A 40-year-old female patient was admitted due to headache. Angiography showed eight aneurysms in the posterior circulation and, bilaterally, in the anterior circulation. All aneurysms were treated endovascularly in two sessions. In the treatment of the aneurysms, different endovascular techniques were used including flow diverters stents, stent-assisted coiling, Y-stent-assisted coiling, and coiling alone.

## 1. Introduction

The frequency of multiple intracranial aneurysms seen in patients with or without subarachnoid hemorrhage (SAH) is high. In many published articles, the rate of multiple intracranial aneurysms has been reported as being between 7% and 45% [1–6]. The advancement of endovascular techniques and devices has ensured that endovascular treatment of intracranial aneurysms is the first choice in most cases, especially for unruptured aneurysms. Surgical treatment of multiple intracranial aneurysms requires multiple craniotomies in most cases. There are some articles reporting the treatment of multiple intracranial aneurysms using 1-stage clipping or coiling, or a combination of both techniques [5–10]. The decision of the best treatment modality should be made utilizing a multidisciplinary approach [7, 8, 11]. In the literature, the maximum number of aneurysms in one patient treated endovascularly and/or surgically is seven [5, 11].

In this case report, we present, with a review of the literature, a patient with eight intracranial aneurysms, all of which were treated in two sessions with various endovascular techniques.

## 2. Case Presentation

**Figure 1:**
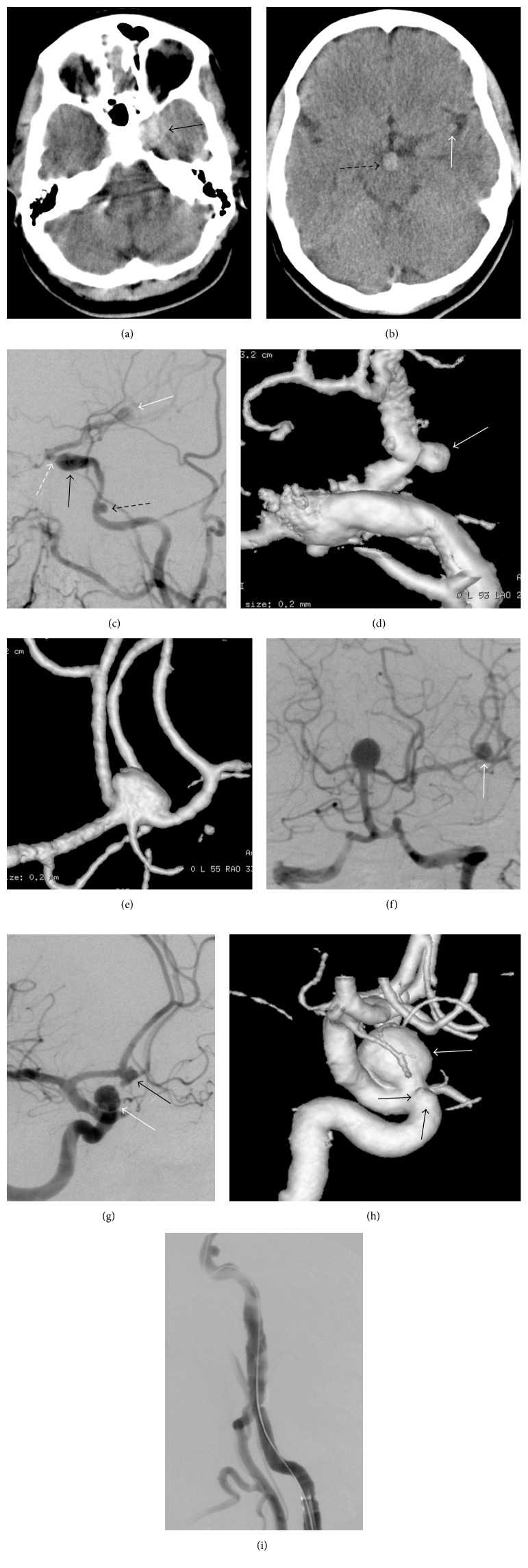
Nonenhanced axial CT slices ((a) and (b)) show three round hyperdensities: one adjacent to the sphenoid corpus on the left, another in front of the mesencephalon, and the third in the Sylvian fissure on the left, implying aneurysms of the internal carotid artery (ICA) cavernous segment (arrow, (a)), basilar tip (dashed arrow, (b)), and middle cerebral artery (white arrow, (b)), respectively. The DSA of the left carotid artery (c) reveals a small wide-necked aneurysm of the ICA lacerum segment (black dashed arrow, (c)), a fusiform aneurysm of the ICA cavernous segment (black arrow, (c)), and a middle cerebral artery bifurcation aneurysm (white arrows, (c) and (f)). A severe stenosis (white dashed arrow, (c)) adjacent to the fusiform aneurysm was also seen, resulting in reduced distal flow (c). 3D images ((d) and (e)) show the small saccular narrow-necked aneurysm of the anterior choroidal artery (white arrow, (d)) and the small saccular wide-necked aneurysm of the middle cerebral artery (e). A saccular wide-necked basilar tip aneurysm (f), a fusiform aneurysm of the anterior communicating artery (black arrow, (g)), and a saccular narrow-necked aneurysm (white arrows, (g) and (h)) and a blister-like (black arrows, (h)) aneurysm of the right ICA ophthalmic segment are seen in the DSA ((f) and (g)) and 3D (h) images. The ICA cervical segment dissection (i) possibly occurred during embolization of the aneurysms.

A 40-year-old female patient was admitted due to headache beginning one month previously and steadily worsening. A nonenhanced CT was performed due to high suspicion of SAH. The CT showed no SAH but round hyperdensities adjacent to the sphenoid corpus on the left, in front of the mesencephalon, and in the Sylvian fissure on the left implied internal carotid artery (ICA) cavernous segment, basilar tip, and middle cerebral artery (MCA) aneurysms, respectively (Figures 1(a) and 1(b)). Angiography was performed. Eight aneurysms were seen in the posterior circulation and, bilaterally, in the anterior circulation (Figure 1).  Table 1 shows the details of the aneurysm locations, characteristics, treatment technique, and stents.

The fusiform aneurysm of the left ICA cavernous segment was partially thrombosed, and there was a severe stenosis at the distal part of the aneurysm (Figure 1(c)). The distal flow beyond the stenosis was reduced significantly. Most of the left MCA blood flow came from the posterior communicating artery (PComA) due to the stenosis (Figure 1(f)).

All aneurysms were decided to be treated endovascularly in two sessions. In the morning, about 8 hours before the procedure, 500 mg of acetylsalicylic acid (Aspirin; Bayer Healthcare, Germany) and two tablets of clopidogrel (Plavix; Bristol-Myers Squibb/Sanofi Pharmaceuticals, NY, USA) were loaded.

After administering general anesthesia, the left common carotid artery (CCA) was catheterized with a long 6F introducer (NeuronMax 6F, Penumbra Inc., Alameda, CA, USA) and the ICA with a 5F distal access guiding catheter (Navien, Covidien AG, Paris, France). After insertion of the latter, 5000 IU heparin was administered IV, targeting two or three times the baseline value, and the serum-activated coagulation time (ACT) was checked. During the procedure, 1000 IU or more of heparin was administered per hour to keep the ACT level stable. After the procedure, 750–1000 IU/h heparin was infused for 24 hours.

Before the treatment of the cavernous segment fusiform aneurysm, the stenosis adjacent to aneurysm was dilated with two Gateway balloons with diameters of 1.5 × 15 and 2.5 × 15 mm (Stryker Neuroendovascular, Kalamazoo, MI, USA). Then, two Surpass flow diverter (FD) (Stryker) stents were implanted to cover the fusiform cavernous and saccular lacerum segment aneurysms. To provide proper apposition of the Surpass stents, a Scepter C balloon (Microvention Terumo, Tustin, CA, USA) with a diameter of 4 × 15 mm was used.

The small anterior choroidal artery aneurysm was embolized with bare coils only, using an Excelsior SL-10 microcatheter (Stryker) and a 0.012 hydrophilic microguide wire with a double-angled tip (Terumo Medical Corporation, Tokyo, Japan). The bifurcation aneurysm of the left MCA was totally closed with stent-assisted (LeoBaby, Balt, Montmorency, France) coiling with a jailed microcatheter, Excelsior SL-10. The left ICA cervical segment dissection, which may have occurred during catheterization, was seen in final angiograms of the left side. The dissection was treated with a carotid Wallstent (7 × 30) (Stryker). Then, the right CCA and ICA were catheterized with the same long introducer and distal access guiding catheter. The fusiform aneurysm of the anterior communicating artery was completely filled with coils, using an Excelsior SL-10 microcatheter. Then, a LeoBaby stent was deployed to prevent recanalization. It was decided that the other aneurysms would be treated in a separate session.

One day later, the patient had no complaint except headache and DWI showed no ischemic lesion. Ten days later, the patient was readmitted to the angiosuite for treatment, under general anesthesia, of the other aneurysms. First, a right ICA DSA performed before starting the treatment showed insufficient apposition of the distal part of the second proximal Surpass stent inside the proximal part of the first Surpass. An attempt to ensure sufficient apposition of the second stent using two Gateway balloons (3 × 15 and 3.5 × 15 mm) was unsuccessful. The stents were left as they were; no further attempts at proper apposition were made.

The patient's basilar tip aneurysm was treated with Y-stent-assisted coiling (Table 1), after enabling access to the left vertebral artery using a 6F NeuronMax with a 6F FargoMax (Balt) distal access guiding catheter. A Neuroform EZ stent (Stryker) was deployed as the first stent from the left posterior cerebral artery to the basilar artery. Following catheterization of the aneurysm with an Excelsior SL-10 microcatheter, an Enterprise stent (Codman Neuro, Raynham, MA, USA) was deployed through a Prowler Select Plus microcatheter (Codman Neuro) and the aneurysm was completely coiled via the jailed Excelsior SL-10.

The ophthalmic segment aneurysm of the right ICA was completely coiled; then, a Surpass stent was implanted to prevent recanalization and to cover the blister aneurysm proximal to the aneurysm of the ophthalmic segment. The proximal part of the Surpass was not apposed to the vessel wall due to its acute curvature. To appose the proximal part of the Surpass stent, a Transform occlusion balloon catheter (5 × 15 mm) (Stryker) was used, but the shape of the proximal part of the Surpass did not change. Then, an Enterprise stent (4 × 22) was deployed inside the Surpass and proper apposition was provided.

The patient was awake and had no neurologic symptoms. DWI was again performed and no ischemic lesion was detected. The patient's only complaint at discharge was of a mild headache. 100 mg of Aspirin was ordered to continue indefinitely and one tablet of Plavix for 6 months.

The 3rd-month follow-up angiograms showed an excellent reconstruction of the cavernous segment aneurysm, as well as disappearance of the petrous segment aneurysm of the left ICA. The anterior choroidal artery (AChorA) aneurysm was still filling but there was no regrowth. The left MCA, anterior communicating artery (AComA), basilar tip, right ICA ophthalmic segment, and right blister aneurysms were fully closed, but there was mild hyperplasia inside the stents used for the MCA, AComA, and basilar tip aneurysms. A slight narrowing was seen inside the distal part of the Surpass stent, due to foreshortening of the Surpass distal part, used for right ICA ophthalmic segment aneurysms. There was no intimal hyperplasia or in-stent stenosis in the left ICA cervical segment dissection treated with Wallstent.

**Figure 2:**
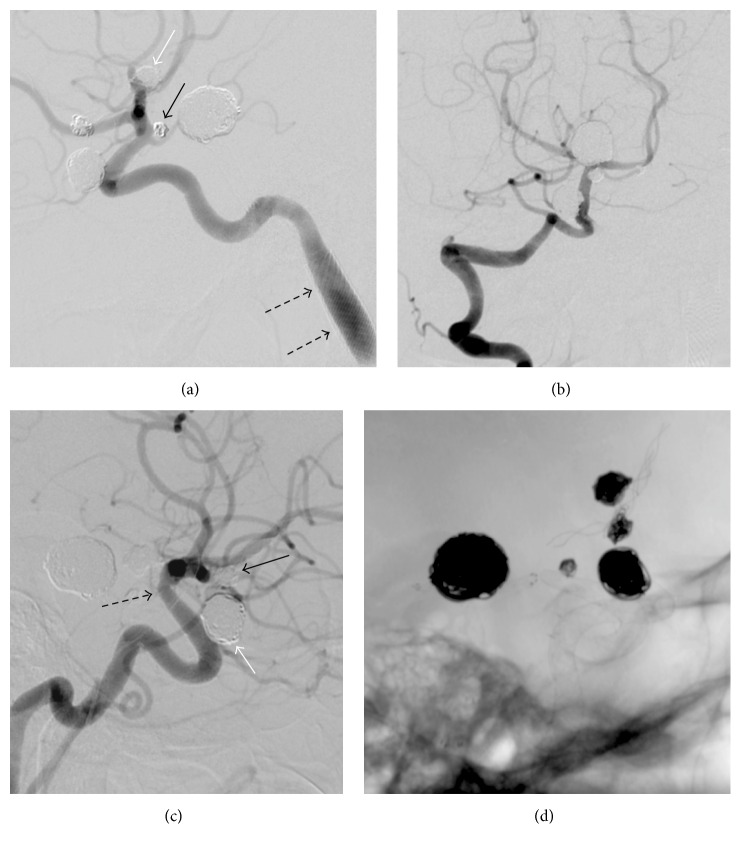
The 9th-month follow-up DSA ((a), (b), and (c)) images show total closure of the aneurysms of the left ICA lacerum segment (a) and the cavernous segment (a), the left anterior choroidal artery (black arrow, (a)), the middle cerebral artery (white arrow, (a)), the basilar tip (b), the anterior communicating artery (black arrow, (c)), and the right ICA ophthalmic segment (white arrow, (c)). The intimal hyperplasia inside the stents seen in the 3rd-month follow-up angiograms (not shown here) was totally relieved. The coils and stents used for the treatment of the aneurysms are seen in the fluoroscopic image (d). A slight fusiform dilation and no in-stent stenosis are seen in the left ICA cervical segment dissection, which was treated with a carotid Wallstent (black dashed arrows, (a)). The 3rd-month DSA (not shown here) showed a slight narrowing, due to the fish mouth or foreshortening effect, inside the distal part of the Surpass stent used for the right ophthalmic segment aneurysms. The 9th-month follow-up DSA (c) showed no change (black dashed arrow, (c)) and no intimal hyperplasia.

The hyperplasia inside the stents was totally relieved and all aneurysms were closed in the 9th-month follow-up angiograms (Figure 2).

## 3. Discussion

When surgery is selected for treatment of multiple intracranial aneurysms, multiple craniotomies are usually required [12]. A single-stage procedure is indicated by multiple intracranial aneurysms on the same side of the anterior circulation. But if the aneurysms are located bilaterally in the anterior circulation or if they are in both the anterior and the posterior circulations, a two-stage procedure is suitable [10]. Hydrocephalus and edema of the brain make access to multiple aneurysms more difficult in the first few days following SAH [1]. Many studies showed clipping of multiple aneurysms results in poorer outcomes than in a single aneurysm [4, 12–14]. This can be explained by the increased manipulation of brain tissue and vasculature during multiple-aneurysm surgery [13, 14]. On the other hand, coiling for multiple aneurysms involves no manipulation of cerebral arteries or of brain tissue. As such, when multiple aneurysms are located either on both sides or in both the posterior and anterior circulations, a single-stage treatment with coiling may be more practical than the one with clipping [6]. In Shen et al.'s [9] study of 84 aneurysms in 36 patients, they chose to leave 19 of the aneurysms untreated. Of the remaining 65, two were treated surgically by clipping and the rest using various endovascular techniques. Their conclusion was that endovascular treatment was a more suitable method than surgery for multiple intracranial aneurysms.

Solander et al. [1] performed endovascular treatment by using Guglielmi detachable coils on 93 aneurysms in 38 consecutive patients and reported the overall clinical outcome was excellent in 34 patients (89%), good in one (3%), fair in one (3%), and fatal in two (5%). Xavier et al. [8] treated the 13 aneurysms of six patients using 1-stage coiling. No periprocedural complications were reported in their study. Jeon et al. [6] reported 1-stage coiling of multiple aneurysms seemed to be safe and effective, with low morbidity and mortality in their study including 167 patients having more than two aneurysms each.

In the literature, searched via PubMed, there is no reported case or study concerning a patient with more than seven simultaneous aneurysms, treated in 1 or more stages either endovascularly, surgically, or by both techniques [1, 5, 7, 8, 10, 15]. In Jeon et al.'s [6] cohort including 167 patients with 359 aneurysms, while two patients had six aneurysms, only one patient had seven aneurysms. They attempted to treat all patients with 1-stage coiling. Oh and Lim [5] treated, with coil embolization in single sessions, 28 patients with multiple intracranial aneurysms. In this group, while only one patient had six aneurysms, the others had less than 6. Ahmed et al. [11] reported a patient with seven aneurysms, one of which ruptured. They treated the patient in two surgical sessions, followed by one endovascular session including only coiling and stent-assisted coiling. In Xavier et al.'s [8] report including six patients, the numbers of aneurysms in each patient were between two and five.

Our patient had eight aneurysms, more than any single patient reported in the literature. The patient also had a severe stenosis adjacent to the left ICA fusiform aneurysm. We treated the aneurysms and the stenosis in two sessions due to their large number. The patient had no SAH, so we used all kinds and combinations of endovascular treatment techniques. We started treating the aneurysms from the left side due to the presence of severe stenosis there. An AComA aneurysm seen during the right side carotid angiography was treated because of its bizarre appearance, with nipples implying imminent rupture. While one fusiform, one saccular, and one blood blister aneurysm were treated only with Surpass flow diverters, two saccular aneurysms were treated with stent-assisted coiling. One wide-necked saccular aneurysm, basilar tip, was treated with Y-stent-assisted coiling, one narrow-necked saccular AChorA aneurysm with coiling alone, and one saccular ICA ophthalmic aneurysm with coiling and flow diverter (Table 1 and Figure 2). The treatment of the patient was accomplished with no complication other than the left ICA cervical segment dissection, which was subsequently treated with carotid Wallstent.

## 4. Conclusion

The rate of multiple intracranial aneurysms is very high and ranges from 7% to 45%. Different combinations of treatment modalities and techniques can be used in the management of multiple aneurysms. But in patients without SAH, treatment of all aneurysms with endovascular techniques in one or more sessions is less traumatic than surgical treatment.

## Figures and Tables

**Table 1:** Aneurysm locations, characteristics, and treatment techniques.

Aneu. no.	Location	Type	Size/neck (mm)	Treatment	Stents	Treat. session
1	Lacerum segment of left ICA	Saccular	3 × 4/4	FD	Surpass 4 × 30	1st
2	Cavernous segment of left ICA	Fusiform	11 × 6	FD	Surpass 4 × 20	1st
3	Left AChorA	Saccular	3 × 3/2	Coil		1st
4	Bifurcation of left middle cerebral artery	Saccular	6 × 4/4	Stent and coil	LeoBaby 2.5 × 25	1st
6	AComA	Fusiform	4 × 5.5	Stent and coil	LeoBaby 2.5 × 25	1st
5	Basilar tip	Saccular	10 × 10/6	Y stenting and coil	Neuroform EZ 3 × 30 and Enterprise 4.5 × 22	2nd
7	Ophthalmic segment of right ICA	Saccular	8 × 8/2.5	FD and coil	Surpass 4 × 20	2nd
8	Ophthalmic segment of right ICA	Blood blister	2.5 × 2.5	FD	Surpass 4 × 20	2nd

AComA: anterior communicating artery, Aneu: aneurysm, AChorA: anterior choroidal artery, FD: flow diverter, ICA: internal carotid artery, and treat: treatment.
